# 

**Published:** 1917-01

**Authors:** 


### SUBJECT INDEX TO VOLUME LXXI

EVENT AND COMMENT

Connecticut's Part in Dental Development, 452.

Conserving Our Drugs, 451.

Conservatism in Practice, 201.

Consolidation of Licensing Boards, 153.

Dedication of Rochester Dispensary, 251.

Errata, 52.

Forsythe Loving Cup, 1.

Journal of National Dental Association, 51.

Legislation for Army and Navy Dental Corps, 351.

Meeting National Dental Assn., 551.

Officers Ohio State Dental Society, 3.

Ohio State Society Meeting, 3.

Our Government in Need of Dental Help, 202.

Our New Dental Corps, 501.

Patriotic Inflammation, 301.

Post Graduate Instruction, 451.

Shall Dentists Enlist or be Drafted, 352.

Should Undergraduate Students Enter the Army, 401.

Standardizing Dental Education, 453.

The Dental Society Habit, 151.

The Ivory Cross, 351.

The Price of an Artificial Denture, 101.

The Profession and the War, 253.

FORMAL PAPERS

A Confidential Word to the Conscientious X-ray Man. By C. N. Johnson, 70.

Address to the Graduating Class, Ohio College of Dental Surgery. By N. S. Hoff, 255.

Advertising that Makes for Ill Health, 26.

A New Operative Procedure Facilitating Prosthodontia. By H. A. Potts, M.D., 56.

Another Step in Higher Medical Education, 40.

A Reasonable Course to Pursue in the Treatment of Pulpless Teeth. By Otto U. King, 512.

A Reasonable Course to Pursue in the Treatment of Pulpless Teeth. By Julio Endelman, 569.

Applying the Dam to Difficult Cervical Cavities. By R. Ottolengui, 516.

A Soldier or Sailor No Better than His Teeth. By Richard Grady, 117.

A Study of Disputed Points in Local Anesthesia. By S. L. Silverman, 59.

Bad Teeth and their Effect on the Laboring Man's Efficiency. By C. E. Smith, 65.

Cancer and the Oral Cavity. By Joseph C. Bloodgood, 224.

Cast Clasps. By N. B. Nesbett, 18.

Cold in the Head, 75.

Current Views Regarding the Tonsils and their Removal, 178.

Dangers of the Bubble Fountain, 33.

Dental Nurses. By K. H. Collings, 575.

Dentistry Versus Medicine from a Physician's Viewpoint. By H. P. Williams, M.D., 328.

Diagnosis and Treatment of Diseases of Dental Pulp. By J. P. Buckley, 552.

Effect of Higher Requirements on Medical College Attendance, 481.

Emetin, A Note of Warning, 79.

Expansion and Contraction of Plaster and Vulcanite. By A. J. Spence, 8.

Fear of Pain and Dental Neglect. By B. J. Cigrand, 368.

Fusospirillary Peridental Gingivitis. By F. E. Taylor, 228.

Grinding the Teeth, Its Cause and Prevention. By C. F. Bodecker, 52.

Gold or Aluminum Linings for Vulcanite Plates, 387.

Habit Culture. By George Van Ness Dearborn, M.D., 524.

Has the Dental Profession Taken Leave of Its Senses? By C. N. Johnson, 206.

Helpful Suggestions for Everyday Practice. By A. H. Merritt, 276.

How High Frequency Violet Ray Effects are Produced. By Geo. H. Reed, 465.

Immediate Root Canal Filling. By C. E. Kells, 113.

Importance of the Care of Children's Teeth. By J. B. Bilderbach, 267.

Investment of the Dentist's Earnings. By C. N. Johnson, 127.

Is Pyorrhea Alveolaris Curable, 354.

Ludwig's Angina. By J. W. Seybolds, 31.

Modern Amalgam Restorations. By J. V. Conzett, 407.

Modern Root Canal Technic Contrasted with College Teaching in 1906. By W. H. Ellis, 310.

Newer Features of Adequate Dietaries, 326.

Observation of Oral Conditions in their Relations to Body Functions. By Waite A. Cotton, 318.

Oral Hygiene and Public Education. By M. W. Rice, 358.

Peridontitis from Pressure Anesthesia, 381.

Preparation of Natural Tooth for a Crown. By F. H. Orton, 373.

Pressure Anesthesia, 182.

Pressure Anesthesia Contra-indicated, 35.

Preventive Dentistry. By R. W. Bunting, 154.

Pyorrhea and Prophylaxis. By F. H. Skinner, 420.

Real Meaning of Fresh Air, 23.

Reaping the Whirlwind, 334.

Recognition. By E. C. Kirk, 565.

Recovery from Heart Failure, 130.

Regional Debility in Riggs' Disease, 302.

Relation of Dental Infection and Dental Diseases. By E. C. Rosenow, 285.

Removal of the Healthy Rather than the Diseased Pulp. By I. N. Broomell, 210.

Report of Committee on Peridontology Nomenclature, 533.

Research as to the Nature of Nitrous Oxide and Ether Anesthesia. By G. W. Crile, M.D., 363.

Retention of Devitalized Teeth without Focal Infection. By M. L. Rhein, 455.

Root Canal Treatment and Filling. By S. M. Hinman, 167.

Should this Tooth be Extracted? 41.

Some New Forms of Orthodontic Mechanism. By E. H. Angle, 29.

Surgical Treatment of Pyorrhea Alveolaris. By Carl D. Lucas, 403.

Swaged Versus Cast Dentures, 473.

The Dentist Who Came Back. By W. W. Eldridge, M.D., 84.

The Early Stages of Dental Caries. By D. E. Caush, 290.

The Effect of Malformation and Infections on the Oral Cavity of the Child. By Stephen Palmer, 119.

The Emetin and Other Treatments of Pyorrhea, 379.

Food Requirements of a Healthy Child, 32.

The Dakin-Carrel Solution. By M. T. Barrett, 237.

The Influence of Dental Diseases on Lactation, 176.

The Menace of Mouth Infections. By Oliver T. Osborn, 503.

The Tariff on Dyestuffs and Drug Prices, 82.

Transmission of Disease by the Sputum, 38.

Treatment and Filling of Root Canals. By E. D. Coaledge, 103.

Treatment of Pyorrhea as a Specialty. By F. C. Pague, 520.

Trench Mouth. By Major F. M. Wells, 470.

The Use of Volatile Irritants in Collapse, 479.

What is Being Successful. By J. F. Connover, 73.

What is Pyorrhea, 36.

Why Drafted Dental Students Should be Permitted to Finish Their Courses, 438.

INDENTS

Abolish Liquors, Doctors Urge, 389.

Absorption of Deciduous Roots, 489.

A Dangerous Injury, 579.

A Dentist's Duty to His Profession, 188.

A Good Practical Hint, 346.

A Hint About Bridges, 337.

A Hint for Partial Lowers, 542.

Aluminum Clasps, 579.

Amalgamated Gold, 389.

Amalgam Filling in the Lungs, 295.

Amputations, 143.

An Old Anesthetic, 590.

Another Hygienist School, 145.

Another German Imposture, 188.

Antidote for Novocain, Suprarenin, 187.

A Peculiar Case, 245, 342.

Applying Devitalizing Paste, 540.

A Protest, 488.

Arthritis and Oral Sepsis, 132.

Automobile Cause of Poliomyelitis, 47.

A Worm Turns, 145.

Bad Advice on Extracting, 137.

Be Thorough, 589.

Bite Testing, 583.

Bridgeport Clinical Report, 185.

Danger of Mouth Spray, 538.

Dental Anesthetic, 338.

Dental Hygienists for Maine, 340.

Dental Hygienist Legislation for Connecticut, 546.

Dental Maternity Clinic, 242.

Dental Operations Concessive, 190.

Dental Operations by Canadian Army, 546.

Dental Uses for Rice Flour, 341.

Doctors of Dental Surgery, 586.

Duty of Society Membership, 491.

Canadian Army Statistics, 242.

Care of the Hands, 184.

Cast Gold Dowels for Crowns, 190.

Cast Gold Crown, 44.

Cellutone for Repairing Models, 190.

Chicago's Dental Week, 184.

Chlorin as a Disinfectant, 243.

Compound Impressions, 585.

Concerning Cancer, 585.

Concerning Pulp Devitalization, 145.

Conserving the Drugs, 495.

Contributing Cause of Cancer of the Throat, 590.

Easy Way to True Carborundum Wheels, 339.

Eat Wisely, 486.

E. C. Kirk Retires, 394.

Effects of Organic Acids of the Teeth, 588.

Esthetic Root Protection, 391.

Estimating the Fee, 345.

Ethyl Chlorid Spray for Pain, 99.

Ethical, What Does It Mean, 547.

Example of a Diet that will Produce Caries, and One that Will Not, 96.

Exemption of Dentists, 187.

Explorer Pickings, 542.

Extracting Abscessed Teeth, 337.

Failure of Bridge Work, 141.

Finishing Amalgam Fillings, 144.

Filing Root Canals Aseptically, 135.

Filling Root Canals, 548.

Fixed Bridges Iniquitous, 131.

Focal Infections, 87.

Food Values, 539.

For Spongy Gums, 581.

Forsythe Dental Library and Museum, 143.

Gold Crown Repair, 187.

Gold or Aluminum Lining for Vulcanite Dentures, 537.

Gutta Percha as a Root Canal Filling, 136.

His Intention, 572.

Hospital Dental Service, 577.

Immediate Replacement of Extracted Teeth, 587.

Important Information about the Dental Protective Association, 97.

Impression Helps, 589.

Increasing Efficiency of Anesthetic Solutions, 586.

Infiltration Anesthesia, 489.

Injection for Conductive Anesthesia, 339.

Inlay Impressions, 241.

Indiana's New Dental Law, 391.

Individual Bases for Dentures, 200.

Insures His Teeth, 487.

Investing and Casting Gold Inlays, 91.

Iodin Lotion, 194.

Ionic Medication for Granuloma and Pyorrhea, 297.

Ipecac in Treatment of Pyorrhea, 536.

Is Clean Bridge Work Practicable, 139.

Limitations of Fixed Bridges, 131.

Magazine Postage, 388.

Make a Survey of the Mouth, 543.

Measures that Protect the Eyes, 346.

Mobility of Bridged Teeth, 141.

Modified Crib Clasp, 142.

Mouth Sepsis, 392.

Mouth Wash, 183.

National Dental Legislation, 397.

Necrobiosis Cause of Pyorrhea, 536.

New Terms, 189.

Noncorrosive Broach, 183.

Optical Glass, 392.

Oral and Dental Surgery for the Army, 494.

Oral Hygiene Regieme, 583.

Our Esteemed Contemporary, 344.

Packing Rubber for Tight Joints, 489.

Pain in the Teeth, 45.

Painless Devitalizing Paste, 296.

Pathologic Root Ends, 490.

Patriotic Work for those Unable to Join the Dental Corps, 390.

Plaster Models, 579.

Platinum, 343.

Plumpers for Vulcanite Plates, 185.

Presumably Going South, 581.

Prevention of Caries, 186.

Principal Defects in Navy Recruits, 395.

Protecting Patients, 493.

Pulp Canal Treatment Errors, 543.

Pulp Devitalization, 489.

Radiographing Root Canals, 540.

Rational Versus Normal Occlusion, 294.

Recementing a Loose Bridge without Removing, 186.

Refining Casting Golds, 490.

Relative Values, 184.

Removing Broken Pin from Detached Post Crowns, 547.

Removing Teeth from Old Dentures, 339.

Repairing Inlay Margins, 43.

Royal Dentistry in Morocco, 384.

Sacredness of a Vital Tooth, 140.

Salesmanship in Dentistry, 590.

Saliva Control, 350.

Saving Pulpless Teeth, 135.

Search for Ideal Antiseptic, 88.

Secure Exact Occlusion, 548.

Should We Extract All Diseased Deciduous Teeth, 245.

Soldering for Accident Repair, 189.

Some Conclusions as to Root Treatments, 89.

Some Thoughts, 338.

So to Speak, 43.

Sterilization of Instruments, 132.

Sterilization of Root Canals, 486.

Sudden Break Down, 341.

Sulpho-Carbolate of Zinc, 585.

Technic for Root Amputation, 581.

Technic of Root Filling, 192.

The Ameba in Pyorrhea, 94.

The Bridge Span, 131.

The Error of Condensing Amalgam, 383.

The Fixed Bridge, 142.

The Ideal Dentist, 143.

The Menace of Diseased Teeth, 541.

The Open Door, 492.

They Knew Him, 142.

Treating Root Perforations, 541.

Treatment of Baldness and Dandruff, 246.

To Make Rubber Tubing Pliable, 339.

To Reduce the High Cost of Bridges, 543.

To Remove Model from Articulator, 389.

Toxemia from Root Infection, 195.

Use for Old Fissure Burs, 185.

Use for Silver Wire, 340.

Use of Suprarenin, 584.

Value of Prophylaxis, 144.

Why are Some Teeth Immune, 186.

Why I Am Making Good, 44.

Why Teeth Decay, 579.

Zinc Oxide and Eugenol Root Filling, 242.

ANNOUNCEMENTS

American Ambulance Hospital, 299.

American Medical Association, 247.

American Institute of Dental Teachers, 198.

American Society of Orthodontists, 347, 396.

A Warning, 591.

Carr Case Decided, 100.

Chicago Dental Society Clinics, 591.

Connecticut State Dental Association, 50, 249.

Dedication Rochester Dental Dispensary, 198.

Examination of Dentists for United States Army, 299.

Four States Convention, 199, 248.

Forsythe Dental Infirmary, 249.

Frank A. Hunter Dined, 196.

Iowa State Board of Examiners, 249, 549.

Illinois State Society, 200.

Illinois State Board of Examiners, 249.

Indiana State Dental Association, 198.

Kansas State Board Examiners, 199.

Kentucky State Association, 50, 200.

Memorial to Dr. Isadore Lett, 199.

Michigan State Board, 249, 446.

Michigan Dental Society, 99, 147.

Minnesota Dental Association, 50, 591.

National Dental Association, 300, 347, 348, 396, 446, 499.

National Association Dental Faculties, 148, 347, 396.

Northern Ohio Dental Association, 200.

Ohio State Dental Society, 549.

Rochester Dental Dispensary, 348.

Tennessee Dental Association, 100, 199, 146.

West Virginia State Society, 99.

BIBLIOGRAPHY

Brady's Dental Metallurgy, 450.
Journal of Military Surgeons, 500.
Oral Roentgenology, 592.

OBITUARY

Henry W. Harvey, 100.

Rudolph H. Hofheinz.

W. A. White.

BIOGRAPHIC

Allen, S. A., 346.

Almon, H, 486, 540, 579.

Angle, E. H., 29.

Anders, J. M., 394.

Arquit, E. P., 145.

Atkinson, L. W., 44.

Atkinson, W. W., 384.

Barrett, M. T., 237.

Bilderbach, J. B., 267.

Black, A. D., 136, 542.

Bloodgood, J. C., 224.

Bodecker, C. F., 52.

Bonar, E. A., 491.

Branson, C. B., 346.

Broomell, I. N., 210.

Brophy, T. W., 139.

Brown, H. C., 400.

Bruun, E. A., 243.

Bryant, L. E., 182.

Buckley, J. P., 137, 194, 559.

Bunting, R. W., 154.

Burgess, J. K., 140, 141, 142, 295.

Butler-Wood, F. G., 340.

Conover, J. F., 73.

Caush, D. E., 290.

Chayes, H. E., 130, 132.

Cigrand, B. J., 368.

Collings, K. H., 575.

Conzett, J. V., 94, 407.

Cooledge, E. D., 103.

Costello, Dr., 396.

Cotton, W. A., 318.

Cozier, M. H., 303.

Crile, G. W., 363.

Crosby, A. W., 97.

Cross, H. A., 337, 543.

Crozier, T. W., 387, 537.

Custer, L. E., 186.

Davis, E. B., 487.

Davis, O. de F., 590.

Dearborn, T. V., 524.

Edgelow, P., 232.

Eldridge, W. W., 84.

Ellis, W. H., 310.

Endelman, J., 569.

Fellman, W. O., 543, 590.

Frahm, F. W., 132, 188, 392, 589.

Gethro, F. W., 545.

Grady, R., 117.

Harkrader, R. C., 390.

Haskins, W. H., 195, 491.

Hinman, S. M., 167.

Hoff, N. S., 255.

Hofheinz, R. H., 48.

Hunter, F. A., 196.

Huschart, J. H., 339.

Jackson, C., 296.

Johnson, C. N., 70, 127, 143, 188, 206, 336.

Jones, Basil, 192.

Kells, E. C., 113, 350.

King, Otto N., 512.

Kirk, E. C., 190, 565.

Kramer, F. B., 489.

Latimer, M. B., 339.

Lockwood, B. F., 583.

Logan, W. H. G., 187.

Low, W. S., 590.

Lucas, Carl D., 403.

Merritt, A. H., 276.

Meyer, F. S., 241.

Moor, W. D., 490.

Moorehead, F. B., 462.

Nesbett, N. B., 18.

Northrop, H. W., 141.

Orton, F. H., 373.

Ottolengui, R., 382, 516.

Osborn, O. T., 503.

Pague, F. C., 520.

Palmer, B. B., Jr., 493.

Palmer, Stephen, 119.

Parker, A., 234.

Pearn, F. C., 541.

Perry, E. J., 548.

Potts, W. A., 56.

Price, W. A., 135.

Prinz, H., 245, 585.

Puterbaugh, P. G., 543, 586.

Pyper, P. A., 184.

Reed, G. H., 465.

Rhein, M. L., 455, 463.

Rice, M. W., 358.

Rolleston, J. D., 236.

Rose, F. A., 389.

Rosenow, E. C., 285.

Schamberg, M. L., 461.

Schamm, H., 338.

Schmitt, J. C., 581.

Semans, H. M., 354.

Seybold, J. W., 31.

Skinner, F. H., 420.

Smith, Carl E., 65.

Shaw, D. M., 142.

Short, W. N., 144.

Silverman, S. L., 59.

Stamper, W. L., 145.

Stephan, F. W., 99.

Spence, S. J., 8.

Stillman, Paul R., 144.

Sturridge, E., 298.

Sutherland, B. S., 186.

Talbot, E. H., 195.

Taylor, F. E., 228.

Thoma, K. H., 464.

Thomas, D. S., 541.

Thornton, A. W., 141.

Thorpe, B. L., 43, 296, 584.

Van Arsdale, A. L., 588.

Wells, F. M., 470.

White, W. A., 550.

Williams, H. P., 328.

Woolgar, C. H., 342.

### ANNOUNCEMENTS

KENTUCKY STATE DENTAL ASSOCIATION? The next Annual Meeting of the Kentucky State Dental Association will be held at Louisville the three days previous to the Kentucky Derby, May 9-10-11, 1917. Address all correspondence to W. M. Randall, secretary, Louisville, Ky.

CONNECTICUT STATE DENTAL ASSOCIATION. This Society will again hold its Annual Meeting in the Griswold Hotel, at New London, the 14th to 16th of June. Papers and clinics of unusual interest, and the excellent accommodations at this beautiful resort hotel will make this a most attractive meeting to attend. For full information address Dr. Geo. S. B. Leonard, secretary, Mystic, Conn.

MINNESOTA STATE DENTAL ASSOCIATION. Will meet February 23-24 in Minneapolis in the Dental Department of the University. Dr. Max E. Ernst, secretary, Lowry Building, St. Paul.

